# Melanoma: implications of diagnostic failure and perspectives

**DOI:** 10.31744/einstein_journal/2021ED6680

**Published:** 2021-12-20

**Authors:** Mara Giavina-Bianchi, Eduardo Cordioli, Birajara Soares Machado

**Affiliations:** 1 Hospital Israelita Albert Einstein São Paulo SP Brazil Hospital Israelita Albert Einstein, São Paulo, SP, Brazil.

Melanoma accounts for only approximately 5% of all skin cancer diagnoses worldwide, but it is very important due to its metastatic potential and the fact that it can lead to death.^(1)^ In Brazil, in 2019, there were 1,978 deaths from the disease, and the estimate was 8,450 new cases for 2020.^(2)^ A recently published study with Brazilian data showed that the mean incidence in men increased from 2.52 to 4.84, and in women, from 1.33 to 3.22 cases/100,000 inhabitants in the period from 2000 to 2013.^(3)^The best management for melanoma remains its early diagnosis and treatment, which is curative in 95% of cases, when located only in skin.^(4)^Failure to early diagnose a case of localized melanoma can bring many dire consequences to the patient. The longer the time lost to diagnosis, the worse the situation becomes. The impact on health care costs by the Brazilian Public Health System (SUS - *Sistema Único de Saúde*) increases exponentially in more advanced cases.

There is not much data on the cost of melanoma in Brazil. A study conducted in 2009 showed 95.8% of all spending in São Paulo state on melanoma was for treatment of stage III and IV patients.^(5)^ This study was carried out when treatment of advanced cases involved only interferon-alpha and dacarbazine. When target therapy and immunotherapy are used, the cost certainly increases, as has been shown in recent studies in Australia and France.^(6,7)^ However, this investment is reflected in the survival of individuals. Ten years ago, the 5-year survival rate of patients with metastatic melanoma was only 10% with chemotherapeutical agents, such as dacarbazine, paclitaxel, and carboplatin. With the new medications, this 5-year survival rate went to 52% for the combination of ipilimumab+ nivolumab (two immunotherapies).^(8)^

On the other hand, in cases where the presumptive diagnosis of skin melanoma is made and the biopsy reveals a benign lesion, such as a melanocytic nevus or seborrheic keratosis, we have a false-positive case. Thus, the individual will have undergone an unnecessary diagnostic procedure, having subjected themselves to surgical risks, scarring, stress of the procedure, loss of workdays, and cost of travel. There are also consequences for the health care system, such as the costs of biopsy and pathology examination, and the unnecessary overload of the system, among others. The number of benign lesions removed for each diagnosis of melanoma varies from 4.5 for experts to 22.3 for non-specialists.^(6)^ Improving diagnostic accuracy is extremely valuable for all involved and for the healthcare system.

How should this challenge be faced? Brazil has a very large territorial extension, and dermatologists, physicians specialized in detecting melanoma early, are heterogeneously distributed throughout the country. According to the last census, 58.9% are in the Southeast, 15.8% in the South, 13.8% in the Northeast, 8% in the Midwest, and 3.5% in the North.^(9)^ Only 9.1% of Brazilian municipalities have dermatologists. This means that people living far from large urban centers have to travel long distances to have access to a specialist. Even in large centers, the demand for dermatologists can be much higher than what the SUS is able to provide, generating a waiting list of up to one year.^(10)^

The use of teledermatology is an excellent option. Evaluation by the specialist, particularly if associated with the use of dermatoscope (teledermoscopy), can avoid many unnecessary surgeries, diagnose true positive cases earlier, and prevent the advancement of melanoma and all the consequences of a late-stage diagnosis.^(11,12)^ Teledermatology also has other advantages, such as convenience for the patient in not traveling to specialized services; high accuracy when compared to face-to-face consults; and costs comparable to or even lower than the traditional form and speed of care. In a project carried out in the city of São Paulo, the use of teledermatology reduced by 78% the average waiting time for an in-person dermatologist appointment, and patients suspected of having skin cancer were already directly referred for biopsy, in an attempt to further shorten the time for diagnosis and treatment.^(10)^

The development of artificial intelligence in the early detection of melanoma is a very current issue and is being developed by many university departments and private companies around the world. Some studies have shown *in-silico* algorithms have proven to have at least the same level of diagnostic accuracy as medical experts.^(13-15)^ Since these algorithms have not yet been put into practice in the real world, the efficiency, applicability, and reliability of, and acceptability by the professionals who will use these resources is not yet known. In any case, one can think of several benefits, which have already been observed in the evaluation of other diseases, such as diabetic retinopathy, stroke, and coronary ischemia.^(16-18)^

One perspective for the use of teledermatology would be in the triage of skin cancer lesions. Artificial intelligence can help to anticipate the diagnosis of melanoma and other very frequent skin cancers, by analyzing nuances of the lesions that might go unnoticed by physicians, especially for non-specialists, thus saving lives and saving resources. In the case of a lesion suspected of malignancy by the artificial intelligence algorithm, the patient would be urgently referred to the dermatologist, so that dermoscopy could be performed, and, with this set of information, the appropriate action could be taken. It is important to emphasize the interpretation of artificial intelligence algorithm results must be done by the physician. In the case that both the dermatologist and the artificial intelligence agree on the diagnosis, the confidence in its management, whether excision or observation, should be greater. In the case of benign lesions that do not need removal, the agreement of opinions could be used to support the physician in convincing the patient who, for fear of cancer, insists on excising the lesion. In situations where there is disagreement between the dermatologist and artificial intelligence, the physician should have the final word. However, considering the disparity of analyses, it would be prudent to suggest re-evaluating the lesion in a short term, *e.g.,* within three months. On the other hand, the use of artificial intelligence through smartphone applications for the lay public should be better studied, because at the same time that it may alert the patient to possible problems with a certain lesion, causing them to seek specialized help, it may also generate unnecessary stress and costs in benign (false-positive) cases.

Although there is great enthusiasm regarding the development of these technologies, there is also a fear on the part of dermatologists that they would replace or significantly change their current situation in the future, reducing the number of consultations, financial remuneration, or even making them obsolete. In principle, this fear is unfounded, since teledermatology aims to offer patients more options and increase their accessibility to dermatologists, not to replace face-to-face consultations. Similarly, the application of artificial intelligence will be incorporated for tracking cases of interest, such as melanoma, and as a diagnostic support system for dermatologists, increasing the accuracy of diagnoses. By updating dermatologists on the limitations and benefits of these technologies, this fear can be reduced. However, many legal and regulatory issues are still open, and it is necessary that these also evolve in parallel, so that both physicians and patients are fully supported.
