# Supplementary material for: Melanoma: implications of diagnostic failure and perspectives
Source: Einstein (Sao Paulo). 2021 Dec 20;19:eED6680. doi: 10.31744/einstein_journal/2021ED6680 (PMC8693881; doi:10.31744/einstein_journal/2021ED6680)
Supplement: Translation

# Translation: Melanoma: implicações da falha diagnóstica e perspectivas

**Authors:** Mara Giavina-Bianchi, Eduardo Cordioli, Birajara Soares Machado

**Affiliations:** 1 Brasil Hospital Israelita Albert Einstein, São Paulo, SP, Brasil.

O melanoma representa apenas cerca de 5% de todos os diagnósticos de cânceres de pele no mundo, porém adquire importância muito grande devido ao seu potencial metastático e de levar o indivíduo ao óbito.^(1)^ No Brasil, em 2019, ocorreram 1.978 mortes pela doença, e a estimativa era de 8.450 casos novos para o ano de 2020.^(2)^ Estudo recentemente publicado com dados brasileiros mostrou que a incidência média em homens aumentou de 2,52 para 4,84 e, nas mulheres, de 1,33 para 3,22 casos/100 mil habitantes no período de 2000 a 2013.^(3)^ A melhor conduta para o melanoma continua sendo seu diagnóstico e tratamento precoces, que é curativo em 95% das vezes, quando o melanoma está localizado somente na pele.^(4)^ A falha no diagnóstico precoce de um caso de melanoma localizado pode trazer muitas consequências ruins para o paciente. Quanto maior o tempo perdido para se diagnosticar, pior se torna a situação. O impacto sobre os gastos com saúde pelo Sistema Único de Saúde (SUS) aumenta exponencialmente nos casos mais avançados.

Não existem muitos dados sobre o custo do melanoma no Brasil. Estudo em 2009 mostrou que 95,8% de todo o gasto no estado de São Paulo com o melanoma foi destinado ao tratamento dos pacientes nos estádios III e IV.^(5)^ Esse estudo foi feito quando o tratamento dos casos avançados envolvia apenas interferon alfa e dacarbazina. No caso de utilização da terapia-alvo e imunoterapia, o custo certamente aumenta, como foi mostrado em estudos recentes na Austrália e na França.^(6,7)^ Entretanto, esse investimento se refletiu na sobrevida dos indivíduos. Há 10 anos, a taxa de sobrevida em 5 anos dos pacientes com melanoma metastático era de apenas 10% com quimioterápicos como dacarbazina, paclitaxel e carboplatina. Com as novas medicações, essa sobrevida em 5 anos foi para 52% para a combinação de ipilimumabe + nivolumab (duas imunoterapias).^(8)^

Por outro lado, nos casos em que a hipótese diagnóstica de melanoma cutâneo for feita e a biópsia revelar lesão benigna, como um nevo melanocítico ou ceratose seborreica, temos um caso falso-positivo. Assim, o indivíduo terá sofrido um procedimento diagnóstico desnecessário, tendo se sujeitado a riscos cirúrgicos, à formação de cicatriz, ao estresse do procedimento, à perda de dias de trabalho e ao custo do deslocamento. Há consequências também para o sistema de saúde, como os gastos da realização da biópsia e do exame anatomopatológico e a sobrecarga desnecessária do sistema, entre outras. O número de lesões benignas removidas para cada diagnóstico de melanoma varia de 4,5 para *experts* até 22,3 para os não especialistas.^(6)^ Melhorar a acurácia diagnóstica é algo extremamente útil para todos os envolvidos e o sistema de saúde.

E como enfrentar esse desafio? O Brasil tem uma extensão territorial muito grande, e os dermatologistas, médicos especialistas em detectar o melanoma precocemente, estão distribuídos de forma heterogênea pelo país. De acordo com o último censo, 58,9% estão no Sudeste, 15,8% no Sul, 13,8% no Nordeste, 8% no Centro-oeste e 3,5% no Norte.^(9)^ Apenas 9,1% dos municípios brasileiros têm dermatologistas. Isso faz com que os moradores distantes dos grandes centros urbanos tenham que percorrer distâncias imensas para ter acesso ao especialista. Mesmo em grandes centros, a demanda por dermatologistas pode ser muito superior ao que o SUS tem capacidade de atender, gerando uma fila de espera de até um ano.^(10)^

O uso da teledermatologia é uma excelente opção, pois a avaliação pelo especialista, especialmente se associada ao uso do dermatoscópio (teledermoscopia), pode evitar muitas cirurgias desnecessárias, além de diagnosticar mais precocemente os casos verdadeiramente positivos e impedir o avanço do melanoma e todas as consequências de um diagnóstico num estadiamento tardio.^(11,12)^ A teledermatologia tem outras vantagens, como conveniência para o paciente em não se deslocar para serviços especializados; alta acurácia quando comparada à presencial; custo comparável ou até inferior à forma tradicional e rapidez no atendimento. Em projeto realizado no município de São Paulo, o uso da teledermatologia diminuiu em 78% o tempo médio de espera para a consulta com o dermatologista presencial, e os pacientes suspeitos de câncer de pele já eram encaminhados à biópsia diretamente, na tentativa de abreviar ainda mais o tempo para o diagnóstico e tratamento.^(10)^

O desenvolvimento da inteligência artificial na detecção precoce do melanoma é um assunto muito atual e vem sendo desenvolvido por muitos serviços universitários e empresas privadas ao redor do mundo. Alguns estudos mostraram que, *in silico*, algoritmos provaram ter, no mínimo, o mesmo nível de acerto diagnóstico que especialistas médicos.^(13-15)^ Como esses algoritmos ainda não foram colocados em prática no mundo real, não se conhece, contudo, a eficiência, a aplicabilidade, a aceitabilidade e a confiabilidade por parte dos profissionais que utilizarão esses recursos. De toda forma, pode-se pensar em vários benefícios, os quais já foram observados na avaliação de outras doenças, como a retinopatia diabética, o acidente vascular cerebral e a isquemia coronariana.^(16-18)^

Uma perspectiva de utilização da teledermatologia seria na triagem de lesões dermatológicas tumorais. A inteligência artificial pode ajudar a antecipar o diagnóstico do melanoma e outros cânceres de pele muito frequentes, ao analisar nuances das lesões que poderiam passar desapercebidas pelos médicos, principalmente para os não especialistas, podendo salvar vidas e poupando recursos. No caso de uma lesão suspeita de malignidade pelo algoritmo de inteligência artificial, o paciente seria encaminhado para o dermatologista com urgência, para que a dermatoscopia fosse realizada, e, de posse desse conjunto de informações, a conduta fosse tomada. É importante ressaltar que a interpretação dos resultados do algoritmo de inteligência artificial deve ser feita pelo médico. No caso de tanto o dermatologista quanto a inteligência artificial concordarem no diagnóstico, a confiança na tomada da conduta, seja exérese ou observação, deve ser maior. No caso de lesões benignas que não necessitam ser removidas, a concordância de opiniões poderia ser usada para amparar o médico no convencimento daquele paciente, que, por medo do câncer, insista em remover a lesão. Já para as situações em que existe divergência entre o dermatologista e a inteligência artificial, o médico deve ter a palavra final, porém, levando-se em conta a disparidade das análises, seria prudente sugerir reavaliar a lesão num curto prazo, como, por exemplo, em três meses. Já o uso da inteligência artificial por meio de aplicativos para *smartphones* para o público leigo deve ser melhor estudado, porque, ao mesmo tempo em que pode alertar o paciente para possíveis problemas com uma determinada lesão, fazendo com que ele procure ajuda especializada, pode também gerar estresse e custos desnecessários nos casos benignos (falso-positivos).

Embora haja um grande entusiasmo com o desenvolvimento dessas tecnologias, há também o medo, por parte dos dermatologistas, de que elas substituiriam ou mudariam significativamente a situação atual deles no futuro, diminuindo o número de consultas, a remuneração financeira ou, até mesmo, tornando-os obsoletos. A princípio, esse receio não tem fundamento, pois a teledermatologia tem como objetivo oferecer mais opções de atendimento ao paciente e aumentar sua acessibilidade aos dermatologistas, e não substituir a consulta presencial. Da mesma forma, a aplicação da inteligência artificial será incorporada para rastreamento de casos de interesse, como melanoma, e como sistema de suporte ao diagnóstico para dermatologistas, aumentando a acurácia dos diagnósticos. Ao se atualizar os dermatologistas sobre as limitações e os benefícios dessas tecnologias, pode-se diminuir esse temor. Existem, no entanto, muitas questões legais e regulamentárias que ainda estão em aberto, fazendo-se necessário que estas também evoluam em paralelo, para que tanto o médico quanto o paciente sejam amparados em sua totalidade.
